# The SaeR/S Gene Regulatory System Induces a Pro-Inflammatory Cytokine Response during *Staphylococcus aureus* Infection

**DOI:** 10.1371/journal.pone.0019939

**Published:** 2011-05-13

**Authors:** Robert L. Watkins, Kyler B. Pallister, Jovanka M. Voyich

**Affiliations:** Department of Immunology/Infectious Diseases, Montana State University-Bozeman, Bozeman, Montana, United States of America; University of California San Francisco, United States of America

## Abstract

Community-associated methicillin-resistant *Staphylococcus aureus* accounts for a large portion of the increased staphylococcal disease incidence and can cause illness ranging from mild skin infections to rapidly fatal sepsis syndromes. Currently, we have limited understanding of *S. aureus*-derived mechanisms contributing to bacterial pathogenesis and host inflammation during staphylococcal disease. Herein, we characterize an influential role for the *saeR/S* two-component gene regulatory system in mediating cytokine induction using mouse models of *S. aureus* pathogenesis. Invasive *S. aureus* infection induced the production of localized and systemic pro-inflammatory cytokines, including tumor necrosis factor alpha (TNF-α), interferon gamma (IFN-γ), interleukin (IL)-6 and IL-2. In contrast, mice infected with an isogenic *saeR/S* deletion mutant demonstrated significantly reduced pro-inflammatory cytokine levels. Additionally, secreted factors influenced by *saeR/S* elicited pro-inflammatory cytokines in human blood *ex vivo*. Our study further demonstrated robust *saeR/S*-mediated IFN-γproduction during both invasive and subcutaneous skin infections. Results also indicated a critical role for *saeR/S* in promoting bacterial survival and enhancing host mortality during *S. aureus* peritonitis. Taken together, this study provides insight into specific mechanisms used by *S. aureus* during staphylococcal disease and characterizes a relationship between a bacterial global regulator of virulence and the production of pro-inflammatory mediators.

## Introduction

*Staphylococcus aureus* predominates as a global cause of bacterial infections, which can range from mild skin irritations to severe life-threatening invasive disease [1]. Increases in reported cases of methicillin-resistant *S. aureus* infections (MRSA) including community-associated MRSA (CA-MRSA) infections that occur in otherwise healthy individuals, independent of hospital settings [2]–[4], are an important public health concern. Generally presenting with soft tissue infections, *S. aureus* disease is also associated with such severe conditions as septicemia, necrotizing pneumonia and necrotizing fasciitis [3], [5]–[8]. In 2005, the United States reported over 18,000 deaths resulting from invasive MRSA disease, a number surpassing the annual fatalities associated with HIV/AIDS [9], [10].

Gram-positive bacterial infections account for ∼50% of all reported sepsis cases and are associated with the dysfunctional production of pro-inflammatory cytokines [11]–[14]. Systemic *S. aureus* infections are associated with the endogenous production of interferon gamma (IFN-γ), tumor necrosis factor alpha (TNF-α) and interleukin (IL)-6 [15], [16]. Additionally, innate immune effectors exhibit severely diminished anti-bacterial function during sepsis and *S. aureus* infections [17]–[21]. However, studies characterizing pathogen-derived mediators of the host inflammatory response have predominately focused on single toxins and proteins, rendering the inflammatory modulating effects of global virulence regulators undefined.

*S. aureus* possesses 16 two-component gene-regulatory systems that monitor changing environmental conditions to influence gene transcription [22]–[24]. Numerous studies have indicated a regulatory role for the *S. aureus* two-component system SaeR/S in the expression of secreted virulence factors [25]–[33]. Previous findings have demonstrated a critical role for *saeR/S* in evading destruction by neutrophils and enhancing mortality in murine bacteremic models [18], [33]. However, significant gaps in our understanding of how *saeR/S* contributes to *S. aureus* pathogenesis exist. To that end, we investigated the influence of *saeR/S* on pathogen survival and the host response during invasive disease and demonstrated that *saeR/S* strongly influenced the production of inflammatory cytokines during *S. aureus* infection. These current findings further support the hypothesis that *saeR/S* is a critical mediator of pathogenesis during staphylococcal disease.

## Results

### *SaeR/S* significantly enhances *S. aureus* survival, dissemination and mortality during invasive disease

**Figure 1:**
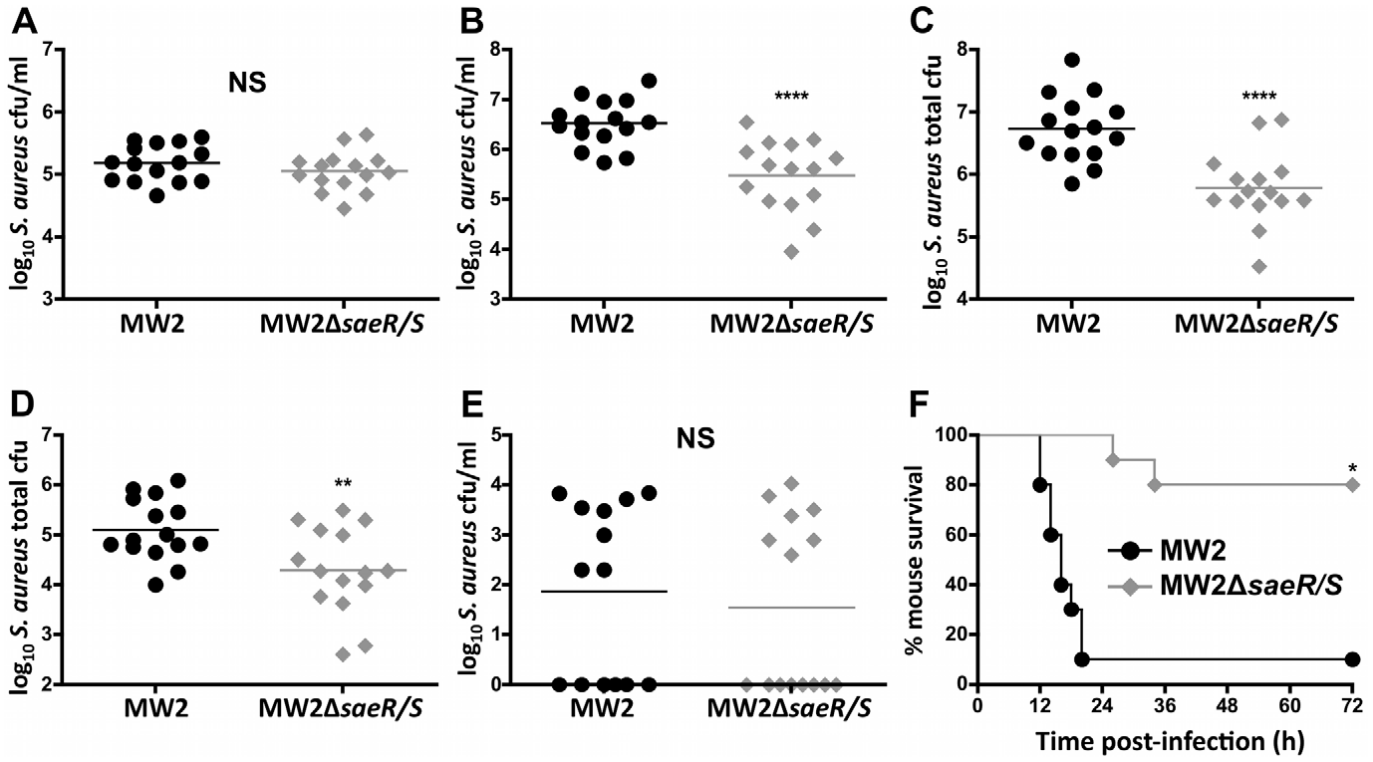
SaeR/S promotes bacterial persistence and host mortality in a mouse model of *S. aureus* peritonitis. Mice were inoculated i.p. with MW2 or MW2*ΔsaeR/S* (5×10^7^ cfu) for (A) 4 hours and (*B*–*E*) 10 hours. (*A*) *S. aureus* burden in the peritoneal exudate, NS. For data in (*A*), results are from 3 biological replicates of 5 mice/group (n = 15). (*B*) *S. aureus* burden in peritoneal exudate, ****P<0.0001. (*C*) *S. aureus* burden in the kidneys, ****P<0.0001. (*D*) *S. aureus* burden in the heart, **P<0.01. *E*, *S. aureus* burden in the blood, NS. For data in (*B*–*E*), results are from 2 biological replicates of 7 and 8 mice/group (n = 15). All tissues compared were individually analyzed by t test. (*F*) Survival curve for mice inoculated (i.p.) with 5×10^7^ cfu of MW2 or MW2Δ*saeR/S*, *P<0.001 as determined by logrank test (n = 10/group). Mice receiving PBS had no *S. aureus* cfu (data not shown). NS = not significant.

Invasive staphylococcal disease is associated with bacterial persistence and dissemination to deep tissues. To investigate the effects of *saeR/S* on bacterial survival and dissemination during invasive infection, we used a mouse model of *S. aureus* peritonitis via intraperitoneal (i.p.) inoculation with wild-type (MW2) or *S. aureus* with deleted *saeR/S* (MW2Δ*saeR/S*) (figure 1). At 4 hours post-infection, *saeR/S* did not impact *S. aureus* survival in the peritoneum, as both MW2- and MW2Δ*saeR/S*-infected mice exhibited similar bacterial loads (*P*>0.05; figure 1*A*). However, at 10 hours post-infection, *saeR/S*-regulated factors significantly increased *S. aureus* survival in the peritoneum of MW2-infected mice (*P*<0.0001; figure 1*B*). To further investigate the contributions of *saeR/S* on *S. aureus* tissue infiltration, we harvested both kidneys and enumerated *S. aureus* burdens at 10 hours post-infection. Mice infected with MW2Δ*saeR/S* had significantly less colony forming units (cfu) in the kidneys (*P*<0.0001; figure 1*C*), suggesting a role for *saeR/S* in pathogen dissemination. To characterize the influence of *saeR/S* on *S. aureus* dissemination from the infectious foci, we exsanguinated mice and harvested hearts for additional *S. aureus* quantification. Consistent with peritoneal cavity and kidney burdens, the absence of *saeR/S* significantly reduced bacterial loads in the hearts from MW2Δ*saeR/S*-infected mice (*P*<0.01; figure 1*D*). Surprisingly, both groups exhibited similar *S. aureus* burdens in the blood (*P*>0.05; figure 1*E*). This suggests a transient presence of *S. aureus* in the blood following peritonitis. Consistent with observations of the influence of *saeR/S* on murine mortality during *S. aureus* bacteremia [18], [33], *saeR/S*-regulated factors significantly enhanced morbidity and mortality in the peritonitis model (1 mouse infected with MW2 survived the 72 hours time course, compared to 8 mice infected with MW2Δ*saeR/S*; *P*<0.001; figure 1*F*). These results are congruent with previous observations that *saeR/S* contributes to host mortality [18], [33] and demonstrates *saeR/S* is essential for *S. aureus* survival and dissemination following invasive infection.

### *SaeR/S* promotes pro-inflammatory cytokine gene transcription

Sepsis syndromes are commonly associated with an early rapid induction of pro-inflammatory cytokines [13]. Following i.p. infection with MW2 and MW2Δ*saeR/S*, we investigated the influence of *saeR/S*-regulated factors on the transcriptional regulation of 84 host-derived inflammation-associated genes (Tables 1, S1 and S2). Leukocytes were isolated from the peritoneal cavity at 4 hours post-inoculation, when the bacterial burdens between MW2 and MW2Δ*saeR/S* were virtually equal (figure 1*A*). Deletion of *saeR/S* resulted in a down-regulation of key inflammatory cytokines (Tables 1 and S1). Of the 84 assayed genes, 47 were down-regulated ≥3-fold with only 6 genes up-regulated ≥3-fold, MW2Δ*saeR/S* relative to MW2 (Tables 1 and S1). Several pro-inflammatory genes commonly expressed during early sepsis were down-regulated in MW2Δ*saeR/S*-infected mice compared to MW2-infected mice, including: IFN-γ: -11.27-fold (*P*≤.05), TNF: -9.37-fold (*P*>0.10), IL-18: −7.17 (*P*≤0.01), CD40 ligand: −4.92 (*P*≤0.01), IL-1β: −4.07 (*P*≤0.05) and C-reactive protein: −4.43 (*P*≤0.05) (Tables 1 and S1). Interestingly, transcription of anti-inflammatory cytokine genes were relatively unaffected with the exception of IL1RII, which was up-regulated 21.38-fold, MW2Δ*saeR/S* relative to MW2 (Table S1). Both MW2 and MW2Δ*saeR/S*-infected mice displayed up-regulation of cytokine transcription compared to PBS-inoculated control mice (Table S2). These results suggest differences in transcriptional activity stem from *saeR/S*-regulated factors, and indicate a *saeR/S*-mediated pro-inflammatory cytokine response during *S. aureus* infection.

**Table 1:** SaeR/S-mediated factors elicit host pro-inflammatory transcription during invasive disease.

Gene symbol	Encoded protein	Fold-regulation change	*P* value
*ifng*	Interferon gamma	−11.27	≤0.05
*tnfrsf1a*	Tumor necrosis factor receptor superfamily, member 1a	−3.03	≤0.01
*il18*	Interleukin 18	−7.17	≤0.01
*cd40lg*	CD40 ligand	−4.92	≤0.01
*il1b*	Interleukin-1β	−4.07	≤0.05
*crp*	C-reactive protein	−4.43	≤0.05
*il11*	Interleukin 11	−3.65	≤0.05

**NOTE.** Genes listed display fold-regulation values of MW2Δ*saeR/S*-infected mice relative to MW2-infected mice (3 per group). RNA was collected from all leukocytes isolated from the peritoneum, 4 hours post-infection. Fold-regulation and *P* values calculated using SA Biosciences™ web-based software utilizing the ΔΔC_t_ method.

### *SaeR/S*-regulated factors promote pro-inflammatory cytokines in the blood during invasive disease

**Figure 2:**
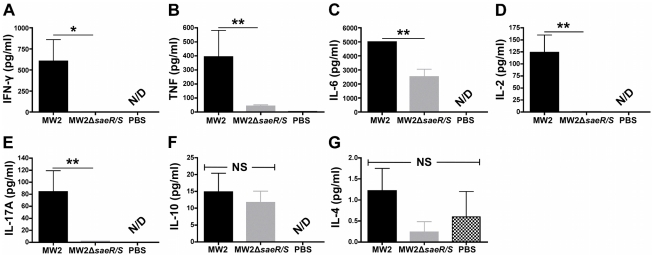
SaeR/S elicits systemic pro-inflammatory cytokines during invasive *S. aureus* infection. Mice were inoculated i.p. with MW2, MW2Δ*saeR/S* (5×10^7^ cfu) or PBS for 10 hours and serum cytokine concentrations were measured by cytometric bead array. Serum concentrations (pg/ml) of (*A*) IFN-γ, (*B*) TNF, (*C*) IL-6, (*D*) IL-2, (*E*) IL-17A, (*F*) IL-10 and (*G*) IL-4. Results displayed are from 5 mice (MW2), 7 mice (MW2Δ*saeR/S*) and 2 mice (PBS). *P<0.05 and **P<0.01 compared to cytokine expression in MW2-infected mice for each cytokine investigated as determined by t test. NS = not significant; N/D = not detectable.

Sepsis and septic shock are associated with the systemic production of inflammatory cytokines [11]–[14]. To determine if the *saeR/S*-mediated inflammatory response was systemic during *S. aureus* peritonitis, we infected mice i.p. with 5×10^7^ cfu of MW2 or MW2Δ*saeR/S* for 10 hours and measured protein levels of several pro- and anti-inflammatory cytokines in the serum by cytometric bead array (figure 2). Of note, serum IFN-γand TNF levels were significantly reduced in mice infected with MW2Δ*saeR/S* (*P*<0.05 and *P*<0.01, respectively; figures 2*A and 2B*). Additional pro-inflammatory cytokines associated with sepsis syndromes were influenced by *saeR/S* as serum IL-6 and IL-2 concentrations were significantly lower in MW2Δ*saeR/S*-infected mice (*P*<0.01; figure 2*C and 2D*). Serum IL-17A levels were also significantly reduced in mice infected with MW2Δ*saeR/S* (*P*<0.01; figure 2*E*). In contrast, IL-4 and IL-10 serum concentrations did not exhibit differences between MW2 and MW2Δ*saeR/S*-infected groups (*P*>0.05; figures 2F and 2*G*). These results are consistent with previous sepsis syndrome observations, in which an early pro-inflammatory cytokine response (IFN-γ, TNF, IL-2, and IL-6) was concomitant with a relatively subdued anti-inflammatory cytokine (IL-4 and IL-10) response [13], [14]. The differentially regulated transcriptional activity in the peritoneum (Tables 1 and S1) and the polarized pro-inflammatory cytokine response in the blood (figure 2) indicate that *saeR/S*-regulated factors promote localized and systemic inflammation during invasive disease.

### Secreted factors, regulated by *saeR/S*, significantly enhance the production of inflammatory proteins in human whole blood

**Figure 3 pone-0019939-g003:**
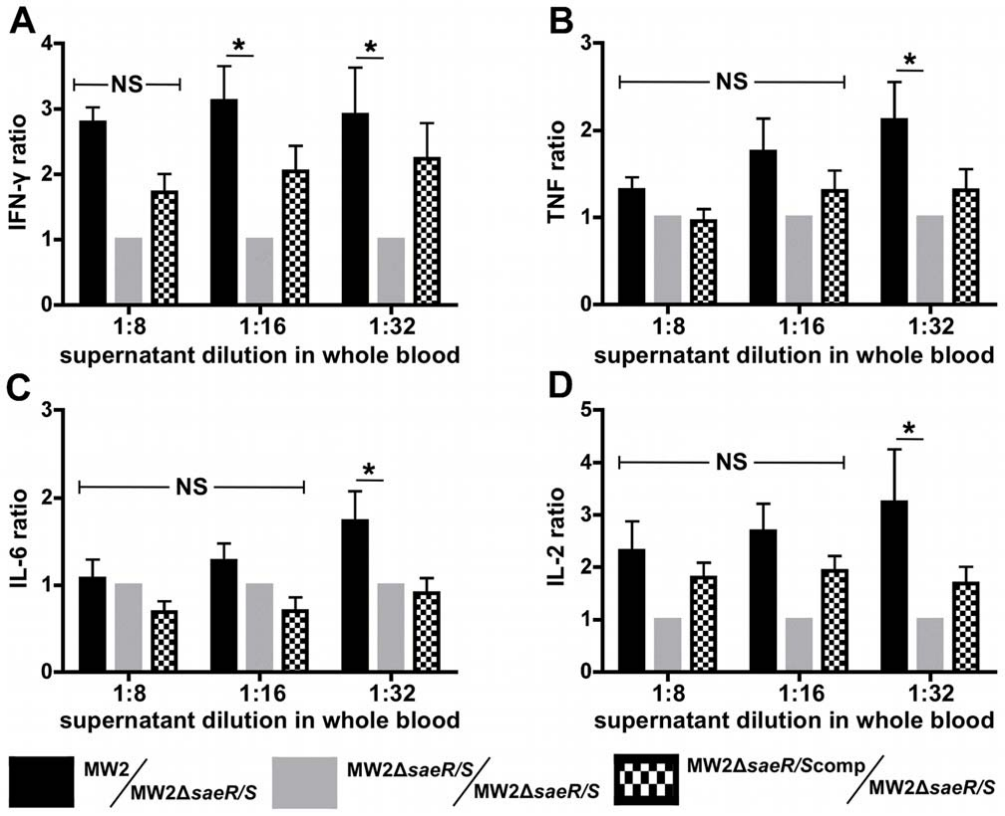
Secreted factors influenced by *saeR/S* promote inflammatory cytokines in human whole blood. *S. aureus* supernatants were collected at early stationary growth phase. Human whole blood was exposed to diluted *S. aureus* supernatants from MW2, *MW2ΔsaeR/*S and *MW2ΔsaeR/*Scomp strains. Cytokine levels were measured after 3 hours. Results represent the blood plasma concentration ratios of (*A*) IFN-γ, (*B*) TNF, (*C*) IL-6 and (*D*) IL-2. Ratios represent the concentration of protein measured in MW2, MW2Δ*saeR/S* or MW2Δ*saeR/S*comp-treated whole blood to the concentration measured in MW2Δ*saeR/S*-treated blood, normalized for each donor. Protein concentrations were measured by cytometric bead array. Results are from 4 separate donors. *P<0.05 versus MW2 as measured by ANOVA with Tukey's post-test. NS = not significant.

**Figure 4:**
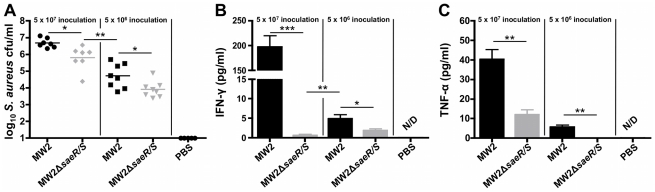
SaeR/S significantly increases IFN-γand TNF-α at the site of infection during invasive disease. Mice were inoculated i.p. with MW2 or MW2Δ*saeR/S* (5×10^7^ or 5×10^6^ cfu) for 10 hours. (*A*) Bacterial burden in peritoneal exudates. Concentrations (pg/ml) of (*B*) IFN-γand (*C*) TNF-α in peritoneal exudates. *P<0.05, **P<0.01 and ***P<0.001 compared to MW2 in each panel as determined by t test. Protein levels for uninfected tissue controls were undetectable. Results are from 7 mice/group for high bacterial infection (5×10^7^ cfu), 8 mice/group for 10-fold reduced bacterial infection (5×10^6^ cfu) and 5 mice in the PBS group. N/D = not detectable.

Numerous secreted *S. aureus* virulence factors are regulated by *saeR/S* [25]–[33]. To investigate the concerted role of these exoproteins on the production of cytokines in human blood and to confirm the *saeR/S*-mediated production of IFN-γ, TNF, IL-6, IL-2 and IL-17A observed in mice (figure 2 and Table 1), we treated human whole blood with supernatants from cultures of MW2, MW2Δ*saeR/S* and a *saeR/S*-complemented MW2Δ*saeR/S* strain (MW2Δ*saeR/S*comp) and compared plasma cytokine levels (figure 3). The data shown are normalized for each individual donor to account for large variations in the magnitude of cytokine expression between individuals. Consistent with cytokine profiles observed in our mouse studies, MW2-treated human blood produced significantly higher levels of IFN-γand TNF compared to MW2Δ*saeR/S*-treated blood (*P*<0.05; figures 3*A and 3B*). Treatment with MW2Δ*saeR/S*comp supernatant restored the observed IFN-γphenotype and partially restored the TNF phenotype (figures 3*A and 3B*, respectively). IL-2 and IL-6 levels were also significantly elevated in the MW2 treatment groups (*P*<0.05; figures 3*C* and 4*D*, respectively), whereas IL–17A was not significantly influenced by supernatants within treatment groups (data not shown). Control groups (media-treated and no treatment) did not result in substantial production of the assayed proteins (data not shown). These results demonstrate that *saeR/S*-regulated secreted factors stimulate the production of pro-inflammatory cytokines in human blood.

### The absence of *SaeR/S* significantly attenuates production of localized IFN-γ and TNF-α production

To further investigate the role of *saeR/S*-mediated factors on the production of IFN-γ and TNF-α at the infectious foci, we measured protein levels in the peritoneal exudates following i.p. inoculation with MW2 or MW2Δ*saeR/S*. At 10 hours post-infection, IFN-γ was significantly reduced in mice infected with MW2Δ*saeR/S* compared to MW2, following i.p. inoculation with 5×10^7^ cfu (*P*<0.001; figure 4*B*). Significant increases in IFN-γ production in MW2 compared to MW2Δ*saeR/S* were also observed using a ten-fold decrease in i.p. inoculum (5×10^6^ cfu; *P*<0.05; figure 4*B*). Of note, mice infected with 5×10^6^ MW2 cfu produced significantly higher IFN-γ concentrations compared to mice infected with 5×10^7^ MW2Δ*saeR/S* cfu (*P*<0.01; figure 4*B*). This demonstrates that differences in bacterial burden (figure 4*A*) do not account for the observed decrease in IFN-γ in MW2Δ*saeR/S*-infected mice and that the robust IFN-γ production is *saeR/S*-mediated. TNF-α protein was also significantly elevated in mice infected with MW2 compared to MW2Δ*saeR/S*, for both 5×10^7^ and 5×10^6^ cfu inoculums (*P*<0.01; figure 2*C*). Collectively, these findings demonstrate that *saeR/S*-regulated factors elicit the production of IFN-γ and TNF-α at the infectious foci during invasive *S. aureus* infection.

### *SaeR/S* promotes IFN-γ during USA300 skin infection

**Figure 5:**
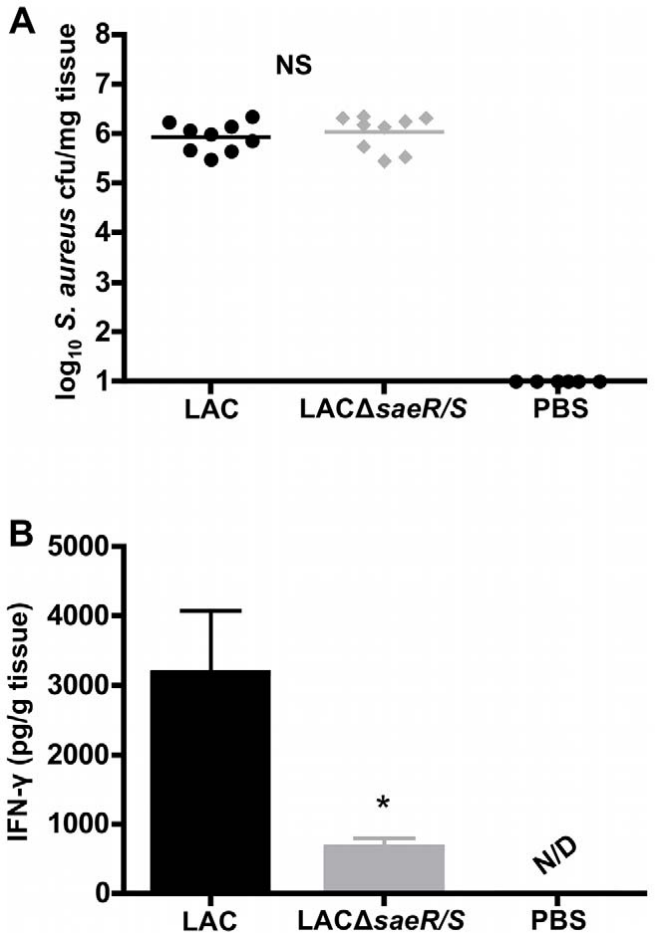
Skin infection with LAC induces IFN-γ in a *saeR/S*-dependent manner. Mice were inoculated subcutaneously with LAC, LACΔ*saeR/S* (1×10^7^ cfu) or DPBS for 8 hours. Infected tissues were excised and homogenized for bacterial load enumeration and IFN-γ concentration measurements. (*A*) Bacterial burden at the site of infection. (*B*) IFN-γ concentration (pg/g tissue) in affected tissues. *P<0.05 compared to MW2 in each panel as determined by t test. Results are from 3 biological replicates of 3 mice/group (n = 9) and 2 mice/PBS group (n = 6). NS = not significant; N/D = not detectable.

*S. aureus* pulsed-field gel electrophoresis type USA300 (LAC) is a major cause of CA-MRSA skin infections [34]. To investigate if *saeR/S* promotes IFN-γ during skin infection and to confirm our previous observation that IFN-γ production is influenced by *saeR/S*-regulated factors, we infected mice subcutaneously with LAC and an isogenic deletion mutant of *saeR/S* (LACΔ*saeR/S*) [33]. At 8 hours post-infection, the deletion of *saeR/S* did not impact bacterial load at the site of infection (Figure 5*A*). These findings are consistent with our previous studies demonstrating *saeR/S* does not significantly influence abscess size or *S. aureus* burden during early skin infection (i.e. less than two days) [18], [33]. However, *saeR/S* did promote a significant increase in IFN-γ in LAC-infected mice compared to LACΔ*saeR/S*-infected mice (*P*<0.05; Figure 5*B*). IFN-γ concentrations in un-infected mouse skin tissues were undetectable (Figure 5*B*). These data are consistent with our observations of enhanced IFN-γ during peritonitis (figure 4) and are in further support that robust IFN-γ production observed during *S. aureus* disease is *saeR/S-*mediated.

## Discussion

In the current study, we found that absence of *saeR/S* significantly decreased the localized and systemic production of pro-inflammatory mediators (i.e. TNF-α, IFN-γ, IL-1β, IL-2, IL-6 and IL-17A) during invasive staphylococcal disease (Tables 1, S1 and figures 2–4). We also observed that robust IFN-γ production was *saeR/S*-mediated during both *S. aureus* peritonitis and superficial skin infections (Table 1 and figures 4 and 5). Significantly elevated systemic pro-inflammatory cytokines coupled with the onset of mortality in wild-type-infected mice strongly indicate that *saeR/S* is critical for mediating sepsis, a phenotype absent in mutant-infected groups. This conclusion is supported by the ‘cytokine storm’ hypothesis, a phenomena characterized by a rapid pro-inflammatory cytokine response that correlates very strongly with coagulation dysfunction, organ failure and death [35], [36]. Our observation demonstrating both pro-inflammatory transcript abundance and protein concentration as significantly elevated in MW2-infected groups (figures 2–4 and Tables 1 and S1), when bacterial burdens are virtually equal at the sites of inflammation (figures 1*A, 1E* and 4*A*), suggest pro-inflammatory cytokine production stems from *saeR/S*-regulated factors. This idea is further supported by our data demonstrating IFN-γ production is significantly reduced in mice infected with MW2Δ*saeR/S* compared to MW2, even when the bacterial burden in mutant *S. aureus*-infected mice is significantly increased (∼10-fold) over wild-type *S. aureus*-infected mice (figures 4*A and 4B*). These data suggest factors regulated by *saeR/S* are responsible for the robust IFN-γ response observed during *S. aureus* infection. However, additional studies are needed to define the contribution of other *S. aureus* global regulators of virulence in mediating the production of IFN-γ and other pro-inflammatory cytokines.

During *S. aureus* infection, the role of IFN-γ has been disputed with studies demonstrating that this inflammatory mediator plays either protective or deleterious roles. For example, using a surgical wound model, McLoughlin et al [37] reported that in the absence of IFN-γ, a decreased *S. aureus* burden was observed at the site of infection. Zhao et al [38] observed that monoclonal antibody-neutralization of IFN-γ decreased the frequency and severity of *S. aureus*-mediated arthritis. Using IFN-γ-deficient mice, Sasaki et al [39] reported a decrease in *S. aureus* burden and an increase in survival rates using a bacteremic model of infection. Conversely, others have demonstrated that administration of exogenous IFN-γ decreased mouse mortality and reduced bacterial loads following *S. aureus* bacteremia [38], [40]. Our current findings correlate increased IFN-γ with elevated bacterial burdens and increased morbidity and mortality. However, it is likely that the observed effects of IFN-γ during *S. aureus* disease are dependent upon multiple factors, including strain of *S. aureus* studied and type/route of infection. Clearly, additional studies are necessary to characterize the precise role of IFN-γ as a mediator of *S. aureus* pathogenesis.

*S. aureus* produces several factors that have been implicated in pro-inflammation, including superantigens and exotoxins [41]. Superantigens non-specifically bind the major histocompatibility complex type II (MHCII) of antigen-presenting cells to T-cell receptors, causing massive T-cell activation and release of pro-inflammatory cytokines [42]. *SaeR/S* regulates several of these factors (18, 33, 43]. For example, Pantrangi et al [43] showed *saeR/S* to positively regulate staphylococcal superantigen-like genes *ssl5* and *ssl8*. Staphylococcal enterotoxin C exhibits a superantigenic phenotype and is also regulated by *saeR/S* [18], [44]. SaeR/S also regulates exotoxin/cytolysin production (i.e. *hla* and *hlg*) and these factors elicit pro-inflammatory cytokines [18], [33], [45]. Finally, our previous study showed that *saeR/S*-regulated factors significantly enhanced the lysis of neutrophils [18]. Cellular lysis promotes the release of host-derived intracellular components and peripheral leukocytes may recognize these danger-associated molecular patterns (DAMPs) to further perpetuate the pro-inflammatory response [46]. Thus, *saeR/S* regulates a full repertoire of factors capable of eliciting a dysfunctional pro-inflammatory cytokine response, an essential mechanism of sepsis.

In addition to *saeR/S*, other *S. aureus* regulatory systems play pivotal roles in virulence and inflammation during infection. Both *agr* and *sarA* are essential for full virulence during invasive *S. aureus* disease [47], [48]. Heyer et al [49] explored the role of *agr*- and *sarA*-mediated cytokine production in a murine model of *S. aureus* pneumonia and reported neither regulator was required for the production of IL-8, a potent pro-inflammatory mediator of neutrophil recruitment. However, both *agr* and *sarA* did promote granulocyte-macrophage colony-stimulating factor (GM-CSF), a cytokine implicated in global pro-inflammatory responses during invasive infection [49]. Furthermore, *agr* has been shown to elicit cytokines and chemokines, important in leukocyte trafficking, from endothelial cells [50]. Taken together with our current report, these studies support the hypothesis that robust host inflammatory responses result from pathogen-derived factors under the influence of global regulators. However, more studies are needed to indentify the specific *S. aureus* factors responsible for the pro-inflammatory response.

In the current study, we report key pro-inflammatory cytokines, associated with sepsis syndromes, as being induced in response to factors regulated by the *S. aureus* two-component gene regulatory system, *saeR/S*. We hypothesize that *saeR/S* plays a critical role in *S. aureus* pathogenesis during invasive disease, likely through a synergistic mechanism of innate immune evasion [25] and the initiation of potentially dysfunctional host inflammatory pathways. This study provides a foundation for future work to identify the individual contributions of specific *saeR/S*-regulated factors to the host inflammatory response during staphylococcal disease.

## Materials and Methods

### Bacterial strains and culture

*S. aureus* isolates, pulsed-field gel electrophoresis type USA400 (MW2) and pulsed-field gel electrophoresis type USA300 (LAC), were selected based on clinical relevance [5]–[11], [17], [18], [33], [51]–[54]. *S. aureus* was cultured in tryptic soy broth (TSB) supplemented with 0.5% glucose and harvested as described elsewhere [17]. MW2, MW2*saeR/S* and MW2Δ*saeR/S*comp were generated in prior investigations [18]. LAC and LACΔ*saeR/S* were generated in prior investigations [33].

### Mouse infection models

All animal studies were performed in accordance with the National Institutes of Health guidelines and approved by the Animal Care and Use Committee at Montana State University-Bozeman. For the peritonitis model, male and female BALB/c mice (aged 8–10 weeks) were purchased from commercial sources and the Montana State University Animal Resource Center. *S. aureus* was harvested at mid-exponential growth phase, washed in sterile Dulbecco's phosphate buffered saline (DPBS) and re-suspended in sterile DPBS at a concentration of 5×10^6^ or 5× cells per 100 µl. All mice were inoculated via intraperitoneal route (i.p.) with MW2 or MW2Δ*saeR/S* and control mice received sterile DPBS.

Bacterial burdens were determined as follows: to enumerate *S. aureus* in the peritoneum, the peritoneal cavity was washed with 10 ml sterile HANKs' balanced salt solution using an 18 gauge needle and 10 ml syringe. Exudate was diluted in distilled water (dH_2_0) and plated on tryptic soy agar (TSA) plates for enumeration of colony forming units (cfu). To determine *S. aureus* load in the blood, mice were anesthetized in isoflurane then exsanguinated via the axillary vessels. Blood was diluted in dH_2_0 and plated on TSA. To determine *S. aureus* burden in selected organs, hearts and both kidneys were aseptically removed, washed in dH_2_0 and then homogenized in dH_2_O. Homogenates were diluted in dH_2_O and plated on TSA. TSA plates were incubated overnight in 37°C; 5% CO_2_ and cfu counted the following day.

The survival study was performed by inoculating mice i.p. with 5×10^7^ *S. aureus*. Mice were monitored every 2 hours for 48 hours and then every 4 hours for an additional 24 hours. Mice were euthanized if they became immobile, exhibited labored breathing or were unable to eat or drink. Survival statistics were performed using a log-rank test.

Skin infection models were performed as described elsewhere [18], [33]. Crl;SKH1-hrBR hairless mice (Charles River) were inoculated subcutaneously with 1×10^7^ bacteria. Eight hours post-bacterial inoculation, the infected skin area was excised using a 9 mm diameter “punch.” Tissues were homogenized in sterile DPBS for bacterial enumeration and cytokine measurements by ELISA per the manufacturer's instructions (R&D Systems).

### Mouse inflammatory gene expression

To compare host inflammatory transcript levels, mice were inoculated i.p. with 5×10^7^ MW2 or MW2Δ*saeR/S* or control DPBS for 4 hours. Peritoneal cavities were washed as described above, and the exudate was centrifuged for 5 min at 600 x g. Pellets were resuspended in RLT lysing buffer (Qiagen) and RNA was purified using RNeasy kits as described by the manufacturer (Qiagen). Contaminating DNA was digested on-column using DNase (Qiagen). Complementary DNA (cDNA) was synthesized using ∼200 ng purified RNA and C-03 RT^2^ First Strand Kit (SA Biosciences). Detection of cDNA was performed using RT^2^ Real-time™ SYBR Green/ROX PCR master mix (SA Biosciences). Master mix with cDNA was loaded onto the Mouse Inflammatory Cytokines and Receptors RT^2^ Profiler™ PCR Array (SA Biosciences). Real-time PCR was performed using a 7500 Fast Real-time PCR system with Fast Real-time PCR system software v1.4.0 (Applied Biosystems). Calculated threshold (C_t_) values were uploaded to the manufacturer's website (SA Biosciences; http://www.sabiosciences.com/pcr/arrayanalysis.php) for analysis using the ΔΔC_t_ method. Fold-regulation and *P* values were calculated by SA Biosciences software (SA Biosciences; http://www.sabiosciences.com). Gene analyses are displayed as fold-regulation values of MW2Δ*saeR/S*-infected mice relative to MW2-infected mice (Tables 1 and S1) and MW2 or MW2Δ*saeR/S*-infected mice relative to DPBS-treated mice (Table S2).

### Mouse cytokine assays

TNF-α and IFN-γ concentrations in peritoneal exudates were measured using sandwich ELISA per the manufacturer's instructions (R&D Systems). Mouse blood was collected as described above, and mouse serum was separated from whole blood by centrifugation. Protein levels of IFN-γ, TNF, IL-6, IL-2, IL-17a, IL-4 and IL-10 were measured using mouse Th1/Th2/Th17 Cytometric Bead Arrays per the manufacturer's instructions (BD Biosciences). All results are displayed as mean concentrations ± SEM (pg/ml).

### Cytokine expression in human whole blood

Heparinzed venous human whole blood was collected from healthy individuals in accordance with an approved protocol by the Montana State University Institutional Review Board. Donors provided written consent to participate in the study. Overnight *S. aureus* cultures were inoculated in TSB at a ratio of 1∶100 and allowed to incubate for 6 hours to early stationary growth phase. At this time, no differences in bacterial growth exist between *S. aureus* strains (data not shown) [18]. Ten ml of *S. aureus* cell suspension was pelleted by centrifugation, and 1 ml of supernatant was sterile filtered using 0.22 µm syringe filters. Filtrate was plated to ensure the absence of *S. aureus* (data not shown). Filtered *S. aureus* supernatant was incubated with 1 ml human blood at final ratios of 1∶8, 1∶16 and 1∶32 using an end-over-end apparatus (Heto Rotamix RK) at 20 RPM for 3 hours at 37°C and 10% CO_2_. Plasma was isolated by centrifugation and stored at −80°C until assayed for protein concentration using human Th1/Th2/Th17 Cytometric Bead Arrays per the manufacturer's instructions (BD Biosciences).

### Statistical Analyses

All data sets were analyzed using GraphPad Prism, version 4.0c for Macintosh (GraphPad Software, San Diego, CA). All mouse data sets were analyzed using paired t tests (see figure legends). Human blood data sets were analyzed using one-way analysis of variance (ANOVA) with Tukey's post-test. For bar graphs, error bars represent the standard error of the mean. Mouse survival statistics were analyzed using the log-rank test.

## Supporting Information

Table S1 Genes listed display fold-regulation values of MW2Δ*saeR/S*-infected mice relative to MW2-infected mice (3 per group). RNA was collected from all cells washed from the peritoneum, 4 hrs post-infection. Fold-regulation values and *P* values calculated using SA Biosciences™ web-based software utilizing the ΔΔC_t_ method.
(DOC)

Table S2 Genes listed display fold-regulation values of MW2 and MW2Δ*saeR/S*-infected mice relative to PBS-treated mice (3 per group). RNA was collected from all cells washed from the peritoneum, 4 hrs post-infection. Fold-regulation values and *P* values calculated using SA Biosciences™ web-based software utilizing the ΔΔC_t_ method.
(DOC)
