# Supplementary material for: The SaeR/S Gene Regulatory System Induces a Pro-Inflammatory Cytokine Response during Staphylococcus aureus Infection
Source: PLoS One. 2011 May 13;6(5):e19939. doi: 10.1371/journal.pone.0019939 (PMC3094403; doi:10.1371/journal.pone.0019939)
Supplement: Table S1 — Genes listed display fold-regulation values of MW2ΔsaeR/S-infected mice relative to MW2-infected mice (3 per group). RNA was collected from all cells washed from the peritoneum, 4 hrs post-infection. Fold-regulation values and P values calculated using SA Biosciences™ web-based software utilizing the ΔΔCt method. (DOC) [file pone.0019939.s001.doc]

Table S1. SaeR/S promotes inflammatory cytokine gene transcription.

| **Gene Symbol** | **Encoded protein** | **Fold-regulation change** | ***P* value** |
| --- | --- | --- | --- |
| *tnf* | Tumor necrosis factor | -9.37 | >0.10 |
| *pf4* | Platelet factor 4 | -2.63 | >0.10 |
| *casp1* | Caspase 1 | -8.21 | >0.10 |
| *c3* | Complement component 3 | -5.39 | >0.10 |
| *il17b* | Interleukin 17B | -2.56 | >0.10 |
| *il4* | Interleukin 4 | 2.14 | >0.10 |
| *il1r2* | Interleukin 1 receptor, type II | 21.38 | >0.10 |
| *il10* | Interleukin 10 | -3.10 | >0.10 |
| *mif* | Macrophage migration inhibitory factor | 2.34 | >0.10 |
| *abcf1* | ATP-binding cassette, sub-family F (GCN20), member 1 | -5.73 | >0.10 |
| *bcl6* | B-cell leukemia/lymphoma 6 | -2.52 | >0.10 |
| *cxcr5* | Chemochine (C-X-C motif) receptor 5 | -5.02 | >0.10 |
| *ccl1* | Chemokine (C-C motif) ligand 1 | -6.65 | >0.10 |
| *ccl11* | Small chemokine (C-C motif) ligand 11 | -7.4 | =0.09 |
| *ccl12* | Chemokine (C-C motif) ligand 12 | -53.84 | >0.10 |
| *ccl17* | Chemokine (C-C motif) ligand 17 | -201.93 | >0.10 |
| *ccl19* | Chemokine (C-C motif) ligand 19 | -4.81 | =0.08 |
| *ccl2* | Chemokine (C-C motif) ligand 2 | -77.42 | >0.10 |
| *ccl20* | Chemokine (C-C motif) ligand 20 | -5.81 | =0.07 |
| *ccl22* | Chemokine (C-C motif) ligand 22 | -4.45 | >0.10 |
| *ccl24* | Chemokine (C-C motif) ligand 24 | 1.55 | >0.10 |
| *ccl25* | Chemokine (C-C motif) ligand 25 | -3.33 | >0.10 |
| *ccl3* | Chemokine (C-C motif) ligand 3 | -9.79 | >0.10 |
| *ccl4* | Chemokine (C-C motif) ligand 4 | -10.69 | =0.09 |
| *ccl5* | Chemokine (C-C motif) ligand 5 | -10.94 | >0.10 |
| *ccl6* | Chemokine (C-C motif) ligand 6 | -28.36 | >0.10 |
| *ccl7* | Chemokine (C-C motif) ligand 7 | -38.19 | >0.10 |
| *ccl8* | Chemokine (C-C motif) ligand 8 | -13 | =0.08 |
| *ccl9* | Chemokine (C-C motif) ligand 9 | -33.14 | >0.10 |
| *ccr1* | Chemokine (C-C motif) receptor 1 | -41.78 | >0.10 |
| *ccr2* | Chemokine (C-C motif) receptor 2 | -1.59 | >0.10 |
| *ccr3* | Chemokine (C-C motif) receptor 3 | 29.29 | >0.10 |
| *ccr4* | Chemokine (C-C motif) receptor 4 | 1.41 | >0.10 |
| *ccr5* | Chemokine (C-C motif) receptor 5 | 17.27 | >0.10 |
| *ccr6* | Chemokine (C-C motif) receptor 6 | 1.82 | >0.10 |
| *ccr7* | Chemokine (C-C motif) receptor 7 | 1.1 | >0.10 |
| *ccr8* | Chemokine (C-C motif) receptor 8 | -2.49 | >0.10 |
| *ccr9* | Chemokine (C-C motif) receptor 9 | -3.22 | >0.10 |
| *cx3cl1* | Chemokine (C-X3-C motif) ligand 1 | -7.53 | >0.10 |
| *cxcl1* | Chemokine (C-X-C motif) ligand 1 | -8.43 | >0.10 |
| *cxcl10* | Chemokine (C-X-C motif) ligand 10 | -4.5 | >0.10 |
| *cxcl11* | Chemokine (C-X-C motif) ligand 11 | -2.74 | >0.10 |
| *cxcl12* | Chemokine (C-X-C motif) ligand 12 | 1.23 | >0.10 |
| *cxcl13* | Chemokine (C-X-C motif) ligand 13 | -1.26 | >0.10 |
| *cxcl15* | Chemokine (C-X-C motif) ligand 15 | -1.8 | >0.10 |
| *cxcl5* | Chemokine (C-X-C motif) ligand 5 | -2.89 | =0.07 |
| *cxcl9* | Chemokine (C-X-C motif) ligand 9 | -3.72 | =0.06 |
| *cxcr3* | Chemokine (C-X-C motif) receptor 3 | -3.31 | >0.10 |
| *ccr10* | Chemokine (C-C motif) receptor 10 | -4.05 | =0.06 |
| *il10ra* | Interleukin 10 receptor, alpha | -5.21 | =0.06 |
| *il10rb* | Interleukin 10 receptor, beta | -3.13 | >0.10 |
| *il13* | Interleukin 13 | -11.88 | >0.10 |
| *il13ra1* | Interleukin 13 receptor, alpha 1 | -3.68 | >0.10 |
| *il15* | Interleukin 15 | -2 | >0.10 |
| *il16* | Interleukin 16 | -2.9 | >0.10 |
| *il1a* | Interleukin 1 alpha | -30.96 | >0.10 |
| *il1f6* | Interleukin 1 family, member 6 | -7.47 | >0.10 |
| *il1f8* | Interleukin 1 family, member 8 | -2.17 | >0.10 |
| *il1r1* | Interleukin 1 receptor, type I | -5.65 | >0.10 |
| *il20* | Interleukin 20 | -3.65 | =0.06 |
| *il2rb* | Interleukin 2 receptor, beta chain | 2.23 | >0.10 |
| *il2rg* | Interleukin 2 receptor, gamma chain | 7.74 | >0.10 |
| *il3* | Interleukin 3 | -7.71 | >0.10 |
| *il5ra* | Interleukin 5 receptor, alpha | -1.12 | >0.10 |
| *il6ra* | Interleukin 6 receptor, alpha | -1.05 | >0.10 |
| *il6st* | Interleukin 6 signal transducer | -3.38 | >0.10 |
| *il8rb* | Interleukin 8 receptor, beta | 3.25 | >0.10 |
| *itgam* | Integrin alpha M | 2.2 | >0.10 |
| *itgb2* | Integrin beta 2 | 1.24 | >0.10 |
| *lta* | Lymphotoxin A | -1.2 | >0.10 |
| *ltb* | Lymphotoxin B | 5.01 | >0.10 |
| *scye1* | Small inducible cytokine subfamily E, member 1 | 1.59 | >0.10 |
| *spp1* | Secreted phosphoprotein 1 | -1.44 | >0.10 |
| *tnfrsf1b* | Tumor necrosis factor receptor superfamily, member 1b | -2.32 | >0.10 |
| *tollip* | Toll interacting protein | -2.19 | >0.10 |
| *xcr1* | Chemokine (C motif) receptor 1 | -2.9 | =0.01 |
