# Supplementary material for: The SaeR/S Gene Regulatory System Induces a Pro-Inflammatory Cytokine Response during Staphylococcus aureus Infection
Source: PLoS One. 2011 May 13;6(5):e19939. doi: 10.1371/journal.pone.0019939 (PMC3094403; doi:10.1371/journal.pone.0019939)
Supplement: Table S2 — Genes listed display fold-regulation values of MW2 and MW2ΔsaeR/S-infected mice relative to PBS-treated mice (3 per group). RNA was collected from all cells washed from the peritoneum, 4 hrs post-infection. Fold-regulation values and P values calculated using SA Biosciences™ web-based software utilizing the ΔΔCt method. (DOC) [file pone.0019939.s002.doc]

**Table S2. Invasive *S. aureus*** infection promotes inflammatory gene transcription.

| **Gene Symbol** | **Encoded protein** | **MW2** | | **MW2Δ*saeR/S*** | |
| --- | --- | --- | --- | --- | --- |
|  |  | **Fold-regulation change** | ***P* value** | **Fold-regulation change** | ***P* value** |
| *abcf1* | ATP-binding cassette, sub-family F (GCN20), member 1 | -1.03 | >0.10 | -5.91 | >0.10 |
| *bcl6* | B-cell leukemia/lymphoma 6 | -1.02 | >0.10 | -2.57 | >0.10 |
| *cxcr5* | Chemochine (C-X-C motif) receptor 5 | -1.75 | >0.10 | -8.77 | >0.10 |
| *c3* | Complement component 3 | 10.39 | >0.10 | 1.93 | >0.10 |
| *casp1* | Caspase 1 | 1.64 | >0.10 | -5.01 | >0.10 |
| *ccl1* | Chemokine (C-C motif) ligand 1 | 4.8 | >0.10 | -1.39 | >0.10 |
| *ccl11* | Small chemokine (C-C motif) ligand 11 | 4.19 | >0.10 | -1.77 | >0.10 |
| *ccl12* | Chemokine (C-C motif) ligand 12 | 78.44 | >0.10 | 1.46 | >0.10 |
| *ccl17* | Chemokine (C-C motif) ligand 17 | 79.39 | >0.10 | -2.54 | >0.10 |
| *ccl19* | Chemokine (C-C motif) ligand 19 | 1.35 | >0.10 | -3.57 | >0.10 |
| *ccl2* | Chemokine (C-C motif) ligand 2 | 19.28 | >0.10 | -4.02 | >0.10 |
| *ccl20* | Chemokine (C-C motif) ligand 20 | 2.85 | >0.10 | -2.04 | >0.10 |
| *ccl22* | Chemokine (C-C motif) ligand 22 | 165.39 | =0.05 | 37.15 | >0.10 |
| *ccl24* | Chemokine (C-C motif) ligand 24 | -10.23 | >0.10 | -6.58 | >0.10 |
| *ccl25* | Chemokine (C-C motif) ligand 25 | 1.12 | >0.10 | -2.96 | >0.10 |
| *ccl3* | Chemokine (C-C motif) ligand 3 | 2570.3 | =0.02 | 262.45 | =0.09 |
| *ccl4* | Chemokine (C-C motif) ligand 4 | 9326.3 | =0.02 | 872.31 | =0.05 |
| *ccl5* | Chemokine (C-C motif) ligand 5 | 4.96 | >0.10 | -2.21 | >0.10 |
| *ccl6* | Chemokine (C-C motif) ligand 6 | 19.6 | >0.10 | -1.45 | >0.10 |
| *ccl7* | Chemokine (C-C motif) ligand 7 | 137.64 | >0.10 | 3.60 | >0.10 |
| *ccl8* | Chemokine (C-C motif) ligand 8 | 6.38 | >0.10 | -2.04 | >0.10 |
| *ccl9* | Chemokine (C-C motif) ligand 9 | 5.73 | >0.10 | -5.78 | >0.10 |
| *ccr1* | Chemokine (C-C motif) receptor 1 | 11.6 | >0.10 | -3.60 | >0.10 |
| *ccr2* | Chemokine (C-C motif) receptor 2 | 2.68 | >0.10 | 1.68 | >0.10 |
| *ccr3* | Chemokine (C-C motif) receptor 3 | -1.73 | >0.10 | 16.92 | =0.01 |
| *ccr4* | Chemokine (C-C motif) receptor 4 | 1.69 | >0.10 | 2.38 | >0.10 |
| *ccr5* | Chemokine (C-C motif) receptor 5 | -2.88 | >0.10 | 5.99 | =0.02 |
| *ccr6* | Chemokine (C-C motif) receptor 6 | 2.67 | >0.10 | 4.86 | >0.10 |
| *ccr7* | Chemokine (C-C motif) receptor 7 | 3.63 | >0.10 | 4.01 | >0.10 |
| *ccr8* | Chemokine (C-C motif) receptor 8 | 6.55 | >0.10 | 2.63 | >0.10 |
| *ccr9* | Chemokine (C-C motif) receptor 9 | 1.29 | >0.10 | -2.49 | >0.10 |
| *crp* | C-reactive protein, pentraxin-related | 1.74 | >0.10 | -2.55 | >0.10 |
| *cx3cl1* | Chemokine (C-X3-C motif) ligand 1 | 10.44 | >0.10 | 1.39 | >0.10 |
| *cxcl1* | Chemokine (C-X-C motif) ligand 1 | 3.42 | >0.10 | -2.47 | >0.10 |
| *cxcl10* | Chemokine (C-X-C motif) ligand 10 | 39.07 | =0.05 | 8.68 | >0.10 |
| *cxcl11* | Chemokine (C-X-C motif) ligand 11 | 18.67 | >0.10 | 6.81 | >0.10 |
| *cxcl12* | Chemokine (C-X-C motif) ligand 12 | -145.96 | >0.10 | -118.76 | >0.10 |
| *cxcl13* | Chemokine (C-X-C motif) ligand 13 | -18.34 | =0.02 | -23.10 | =0.01 |
| *cxcl15* | Chemokine (C-X-C motif) ligand 15 | 1.84 | =0.02 | 1.02 | >0.10 |
| *pf4* | Platelet factor 4 | -5.77 | >0.10 | -15.16 | =0.09 |
| *cxcl5* | Chemokine (C-X-C motif) ligand 5 | 298.09 | >0.10 | 102.98 | =0.01 |
| *cxcl9* | Chemokine (C-X-C motif) ligand 9 | 250.98 | >0.10 | 67.48 | =0.04 |
| *cxcr3* | Chemokine (C-X-C motif) receptor 3 | -2.44 | >0.10 | -8.07 | >0.10 |
| *ccr10* | Chemokine (C-C motif) receptor 10 | -1.38 | >0.10 | -5.58 | >0.10 |
| *ifng* | Interferon gamma | 238.03 | >0.10 | 21.12 | >0.10 |
| *il10* | Interleukin 10 | 122.71 | >0.10 | 39.63 | =0.01 |
| *il10ra* | Interleukin 10 receptor, alpha | 62.63 | >0.10 | 12.03 | =0.01 |
| *il10rb* | Interleukin 10 receptor, beta | 15.03 | >0.10 | 4.80 | >0.10 |
| *il11* | Interleukin 11 | 1.79 | >0.10 | -2.04 | >0.10 |
| *il13* | Interleukin 13 | 32.84 | >0.10 | 2.77 | >0.10 |
| *il13ra1* | Interleukin 13 receptor, alpha 1 | 7.26 | >0.10 | 1.98 | >0.10 |
| *il15* | Interleukin 15 | 3.25 | >0.10 | 1.62 | >0.10 |
| *il16* | Interleukin 16 | -3.46 | >0.10 | -10.03 | >0.10 |
| *il17b* | Interleukin 17B | 1.79 | >0.10 | -1.43 | >0.10 |
| *il18* | Interleukin 18 | 1.09 | >0.10 | -6.58 | >0.10 |
| *il1a* | Interleukin 1 alpha | 126.32 | >0.10 | 4.08 | >0.10 |
| *il1b* | Interleukin 1 beta | 4778.88 | >0.10 | 1173.14 | =0.01 |
| *il1f6* | Interleukin 1 family, member 6 | 54.22 | >0.10 | 7.26 | >0.10 |
| *il1f8* | Interleukin 1 family, member 8 | 1.79 | >0.10 | -1.21 | >0.10 |
| *il1r1* | Interleukin 1 receptor, type I | 2.85 | >0.10 | -1.98 | >0.10 |
| *il1r2* | Interleukin 1 receptor, type II | 19.77 | =0.05 | 422.65 | =0.01 |
| *il20* | Interleukin 20 | 1.79 | >0.10 | -2.04 | >0.10 |
| *il2rb* | Interleukin 2 receptor, beta chain | -2.62 | >0.10 | -1.18 | >0.10 |
| *il2rg* | Interleukin 2 receptor, gamma chain | -14.18 | >0.10 | -1.83 | >0.10 |
| *il3* | Interleukin 3 | 3.05 | >0.10 | -2.53 | >0.10 |
| *il4* | Interleukin 4 | 69.44 | >0.10 | 148.47 | =0.10 |
| *il5ra* | Interleukin 5 receptor, alpha | -8.36 | >0.10 | -9.38 | >0.10 |
| *il6ra* | Interleukin 6 receptor, alpha | -1.98 | >0.10 | -2.08 | >0.10 |
| *il6st* | Interleukin 6 signal transducer | -1.33 | >0.10 | -4.49 | >0.10 |
| *il8rb* | Interleukin 8 receptor, beta | 138.79 | >0.10 | 451.34 | >0.10 |
| *itgam* | Integrin alpha M | 4.17 | >0.10 | 9.19 | >0.10 |
| *itgb2* | Integrin beta 2 | -1.6 | >0.10 | -1.29 | >0.10 |
| *lta* | Lymphotoxin A | 1.45 | >0.10 | 1.22 | >0.10 |
| *ltb* | Lymphotoxin B | -3.45 | >0.10 | 1.45 | >0.10 |
| *mif* | Macrophage migration inhibitory factor | 10.5 | >0.10 | 24.58 | >0.10 |
| *scye1* | Small inducible cytokine subfamily E, member 1 | -1.22 | >0.10 | 1.30 | >0.10 |
| *spp1* | Secreted phosphoprotein 1 | 1591.67 | >0.10 | 1103.54 | >0.10 |
| *tgfb1* | Transforming growth factor, beta 1 | 68.75 | >0.10 | 56.63 | >0.10 |
| *tnf* | Tumor necrosis factor | 367.75 | >0.10 | 39.23 | >0.10 |
| *tnfrsf1a* | Tumor necrosis factor receptor superfamily, member 1a | 322.6 | >0.10 | 106.59 | >0.10 |
| *tnfrsf1b* | Tumor necrosis factor receptor superfamily, member 1b | 195.94 | >0.10 | 84.51 | >0.10 |
| *cd40lg* | CD40 ligand | 21.23 | >0.10 | 4.32 | >0.10 |
| *tollip* | Toll interacting protein | 2.89 | >0.10 | 1.32 | >0.10 |
| *xcr1* | Chemokine (C motif) receptor 1 | 8.9 | >0.10 | 3.07 | >0.10 |
